# When end of treatment situations challenge patient-centered care: a discussion paper proposing new theoretical insights

**DOI:** 10.3389/fpsyg.2024.1445082

**Published:** 2024-10-17

**Authors:** Federica Bonazza, Giulia Lamiani, Lidia Borghi, Silvia Del Negro, Daniela Leone, Elena Vegni

**Affiliations:** ^1^Department of Health Sciences, University of Milan, Milan, Italy; ^2^Department of Psychology, Catholic University of Milan, Milan, Italy; ^3^Unit of Clinical Psychology, San Paolo Hospital, ASST Santi Paolo e Carlo, Milan, Italy

**Keywords:** patient-centered medicine, end-of-treatment, decision-making process, shared decision-making, physician-patient relationship

## Abstract

**Introduction:**

According to the Institute of Medicine patient-centered medicine is one of the six crucial dimensions of health care quality. Although the patient-centered care model is widely recognized for its ethical underpinnings and effectiveness, its practical implementation still raises challenges, especially in end-of-treatment situations. This discussion paper offers an overview of the challenges facing the physician-patient relationship in end-of-treatment situations.

**Methods:**

We developed three clinical vignettes and made some theoretical considerations about ethical issues related to the decision-making process leading to the end of treatment.

**Results:**

We identified two main challenges that end-of-treatment situations pose to patient-centered care: (1) when the patient’s autonomy challenges the best clinical treatment; and (2) when the proposed treatment (discontinuation of treatment) challenges the patient’s preferences.

**Discussions:**

Patient-centered care supports personalized decision-making, in which the physician’s approach varies according to the patient’s situation and individuality. The idea of beneficence may change during care, because of acceptance of the patient’s principles or a change in the primary goal of care.

## 1 Introduction

The Institute of Medicine defines patient-centered medicine as one of the six crucial dimensions of quality healthcare (Hashim, 2017). In contrast to the traditional disease-centered model, patient-centered medicine is a model of care that integrates the biological dimension with the patient’s illness experience and aims to provide care that is tailored to the patient’s individuality including the patient’s values and needs (Moja and Vegni, 2000). Patient-centered care encompasses several components, such as considering the patient’s illness experience, handling the emotions related to the illness, and promoting the patients’ active role in the decision-making process (Lamiani et al., 2008; Bertakis and Azari, 2011). Over the last decade, the patient-centered model has become the gold standard for quality care as application of the clinical method has shown several benefits for patients and their care process, including better health outcomes, greater adherence to treatment, improved patient satisfaction, and better psychosocial adjustment (Bertakis and Azari, 2011; Ekman et al., 2012).

At the ethical level, a core feature of patient-centered care is the importance given to patient autonomy. Autonomy is identified as one of the six values of medical ethics, along with the principles of beneficence, non-maleficence, confidentiality, truth-telling, and distributive justice (Beauchamp and Childress, 2001; Thompson, 1979; Zolkefli, 2018). The value of autonomy encompasses the freedom of an individual to behave in accordance with a plan of their own choosing, act in line with their own desires and ideals, and not being subject to external control (Varelius, 2006). In the health care context, autonomy presupposes that patients should be involved in decision-making regarding their health and express their values and preferences about treatment options. The emphasis on the value of patient’s autonomy is in contrast with paternalism (Emanuel and Emanuel, 1992). Paternalism is a medical approach that assumes that physicians should be the primary decision maker because they have the knowledge to make the best decision regarding the patient’s health (Beauchamp and Childress, 2001; Chin, 2002). The paternalistic approach entitles physicians to limit the patient’s role in the decision-making process and independently make decisions based on what they discern to be in the patient’s best interests, even when patients can make decisions for themselves (Murgic et al., 2015). Over the last twenty years, patient-centered medicine has made many critiques of paternalism and promoted a more active role of patients in their care process, emphasizing the value of autonomy (Komrad, 1983). Respect for patient autonomy is currently the dominant ethos in healthcare (Chin, 2002) and is the basic premise of Shared Decision-Making (SDM). SDM is a process in which physicians and patients work together to reach a decision regarding care. Throughout the SDM process, clinicians and patients work together to clarify treatment, sharing information about the risks, benefits, possible consequences of different options and preferred outcomes with the aim of reaching mutual agreement on the best course of action (Coulter and Collins, 2011). Patient-centered care recognizes the value of autonomy and demonstrates the centrality of shared decision-making, during all the phases of the care process, also at the end-of-treatment.

Although the patient-centered care is widely recognized for its ethical foundations and efficacy (Murgic et al., 2015), its practical implementation still raises challenges and can potentially lead to ethical conflicts (Hansson and Fröding, 2021). Ethical conflicts occur when one ethical obligation appears to conflict with another (Davis, 1990). As far we know, several previous studies shed lights on the potential ethical conflicts resulting from the application of patient-centered care (Davis, 1990; Munthe et al., 2012; Hansson, 2018) but more attention is needed for end-of-treatment situations. The end-of-treatment does not necessarily coincide with the end of life but refers to situations in which it is necessary to stop treatment due to medical conditions or patients’ needs and choices. End-of-treatment situations force patient-centered care to consider important ethical conflicts and challenges, that needed to be theoretically and practically addressed.

In this paper, we provide a reflective discussion on the challenges that the physician-patient relationship within a framework of patient-centered care undergoes in end-of-treatment situations. Specifically, we propose to discuss the implications on clinical decision-making process and the physicians’ role. We identified two main challenges that end-of-treatment situations pose to patient-centered medicine: (1) when patient’s autonomy challenges best clinical treatment; and (2) when proposed (discontinuation of) treatment challenges patient’s preferences. We will discuss these challenges adopting a bottom-up approach. First, three clinical scenarios developed from the authors’ clinical experience as clinical psychologists will be presented. Then, we will make some theoretical considerations about ethical issues embedded in the decision-making process leading to the end-of-treatment will be specifically discussed.

## 2 Self-determination in end-of-treatment situations: when patient’s autonomy challenges best clinical treatment

Vignette 1 presents an exemplary situation in which the patient’s clinical decision is in contrast with the treatment option proposed by the medical team. It is informed refusal, as the patient refused the treatment despite it being suggested by physicians as “best for his health.” In this scenario, patient autonomy is demonstrated by his decision to behave in accordance with his own choices and values even when it means declining medically indicated treatment. The patient’s preferences were expressed in the Advanced Treatment Directives document and confirmed during hospitalization, resulting in the patient’s choice to refuse essential treatments. The patient’s choice forced physicians to withhold treatment and consequently witness the patient’s death.

**Vignette 1: O.**

O. is about 80 years old. He has been admitted to the Intensive Care Unit (ICU); the elective treatment for the patient is endovascular aneurysm repair (EVAR) with endoprosthesis. During the first postoperative day, the patient suffered from axillary bleeding and was treated with surgical hemostasis, but surgery resulted in spinal cord ischemia with paraplegia. The clinical situation has become complicated as the patient has presented with pneumonia. Consulting the patient’s medical record, the document related to the patient’s advanced treatment directives was found and discussed. The document had been compiled 3 years earlier in the municipality of residence. In consideration of this document, sedation had to be reduced to inform the patient of the onset of disability and the possibility of undergoing tracheostomy. Sedation was reduced until the patient was conscious and communication was possible. He was informed of the outcomes of the hemorrhage and the need for tracheostomy for a pulmonary infection. The patient was able to discern and declared that he wanted to be extubated, refusing a tracheostomy, even though aware that this choice would result in worsening of his condition and death. In a second interview the patient reiterated his preference and consented to sedation only, refusing any other treatment. After a discussion among the intensive care team, surgeons and the medical examiner, the patient was extubated. O. died after a few hours.

When patient autonomy is not aligned with proposed clinical care, a discrepancy occurs between the choice and the physicians’ chosen options (e.g., tracheostomy, antibiotic, re-evaluation with rehabilitation purpose). The intervention proposed by the physicians would require treatments, such as tracheostomy, contrary to the patient’s preferences and values. The patient’s final choice led to the avoidance of treatment: in the doctor-patient relationship the decisional balance hangs toward the patient. Legislation and the ethical value of autonomy required physicians to accept a patient’s choice, even in a life-threatening condition that would be reasonably treatable.

Such situations constituted an ethical conflict from the physicians’ perspective. The ethical value of beneficence comes up against autonomy and self-determination, which are predominant. Hence, there is tension between the responsibility to act in the patient’s best interest and the obligation to respect the patient’s autonomy. In other words, in situations like this, respecting patient autonomy requires the physician to renounce the first and most important principle of the Hippocratic Oath, namely, the obligation to act to obtain the patient’s good. Respect for the value of autonomy, in this scenario, implies renouncing pursuit of the value of beneficence and entails a moral conflict for physicians. As a result, physicians have changed their role from that of providing care and treatment to that of accompanying patients in a decision leading to the end of life. Although not being able to provide appropriate care may lead physicians to disengagement (Seeman and Seeman, 1983), they should continue patient care, by accompanying the patient through the sequelae determined by his/ her choice. The physician’s role does not end with the decision but continues by supporting the patient in managing the predictable outcomes of the choice (Rodriguez-Osorio and Dominguez-Cherit, 2008).

Lastly, end-of-treatment situations such as the one described push physicians toward an informative, active, and respectful dialogue with the patient. Physicians must verify that the choice is informed and legitimize patients’ decisions, even if they differ from their own or from those that would guarantee clinical benefits.

## 3 Palliative paternalism in end-of-treatment situations: when proposed (discontinuation of) treatment challenges patient’s preferences

Vignette 2 presents the vignette of Ms. A and Mr. G who underwent an Assisted Reproductive Technology (ART) pathway due to infertility. In the ART setting, there is no clear and univocal medical criteria determining the end-of-treatment, but the end-of-treatment occurs or should occur “when the chance of success is so low that it is in the couple’s best interest to discontinue the treatment” (Boivin et al., 2005). The lack of criteria for treatment interruption makes the decision to discontinue ART particularly complex for the physician-patient relationship.

**Vignette 2: Ms. A. and Mr. G**

A. A 42-year-old woman, suffering from endometriosis since the age of 24. She has a degree in veterinary medicine and works at a clinical center. G, 44, an agronomist, manages a facility that produces organic wines. They are an infertile couple who came to the ART center after a series of 6 IVF attempts at first at a public facility, and later at a private, contracted center. The last attempt was done with heterologous (egg donation), but the embryo did not implant. The probability of a new cycle of egg donation being successful is less than 1%. The couple reports that the last 3 years (after a first year and a half before the infertility diagnosis) have been a real strain, and G. is particularly concerned for A. as the last failure took a lot out of her physically and psychologically. They think, however, that “since they are a good couple, one should not give up”. Therefore, they requested a new consultation at another center. The consultation with the doctor focused on the choice of assisted reproductive technology (ART) in old age and the difficulties associated with it. G. asked the doctor to be frank and help them with the choice that independently they are unable to make. The doctor then explained the age-related problem, risks, and costs. He emphasized that he does not want to decide for them but does not give willingness to continue with new treatment due to the many difficulties. Given this perspective, the couple decides to go to another ART center to pursue treatment.

This scenario is exemplary of many complex situations in which there is still the possibility of continuing treatment, but the probability of success is so low that the persistence of treatment only results in an unnecessary physical and psychological burden. In this scenario, patient autonomy is expressed in the choice to continue treatment despite the doctor’s perspective and recommendation.

These situations can easily lead to overtreatment during clinical care. For “overtreatment” we intend starting or continuing medical procedures that have no other aim but to prolong the patient’s hope (Lepine and Pazos, 2007; Leone et al., 2022). In a condition in which a patient and a physician persist in treatments even though the possibility of a positive impact is significantly doubtful, the decision-making process is particularly complex. Who will finally take the responsibility for ending treatment and how will the process unfold?

Over the past decades, several authors have argued that end-of-treatment situations at risk of excessive persistence may benefit from a new paternalistic style called palliative paternalism (Roeland et al., 2014). Paternalism is a model of traditional medicine. This model allows physicians to intervene and overrule patient’s preferences to respect one of the oldest and most essential principles of the medical profession: the value of beneficence (Beauchamp and Childress, 2001; Chin, 2002). Paternalism has been rejected with the rise of patient-centered medicine and the emphasis on personal autonomy. However, Roeland et al. (2014) introduced the concept of “palliative paternalism”. Palliative paternalism indicates a medical approach that aims to balance the appropriate level of patient autonomy with the physician’s professional judgment to avoid non-beneficial treatments (Roeland et al., 2014). Palliative paternalism differs from traditional paternalism since the physician uses informed options to guide patients in making choice, reduces disinformation, and prevents futile treatment. Furthermore, palliative paternalism includes understanding the likelihood of the patient not making a decision and carrying on the responsibility to recommend the end of unsuccessful treatment (Roeland et al., 2014; deBlois, 1994). Palliative it paternalism can be considered essential, particularly in end-of-treatment conditions, as it helps prevent overtreatment situations, when patients persist with futile treatments that offer no benefits. However, several studies have shed lights on its drawbacks, including the risk of undermining patient autonomy by prioritizing the physician’s perspective, the risk to impose physicians’ values over the patients’ preferences, and the risk of conflict between patients/families and physician in case of disagreement on the best course of action (Bailoor et al., 2018).

To date, the concept of palliative paternalism can be revised in accordance with the SDM process. Under end-of-treatment conditions, a partnership is necessary that requires both parties to negotiate a solution that is acceptable to both parties. Physicians should demand a favorable risk–benefit ratio for patients from the intervention. In circumstances such as Ms. A and Mr. G’s vignette, there is leeway to continue treatment, but the cost to the patient would be significant. Continuing treatment may meet the patient’s preference and autonomy, but it may also involve abandoning the physician’s professional obligations related to beneficence and non-maleficence values, with the risk of administering therapies to the patient that are no longer appropriate (Boivin et al., 2005). In the scenario 2, the physician exercises palliative paternalism with negative outcomes. Although the physician respects the value of beneficence by not continuing the patients’ desired treatment and opposing their preferences, the couple decides to persist elsewhere, demonstrating a lack of understanding of the physician’s perspective. Patients may choose to persist with treatment, for example seeking availability from other doctors and starting the “doctor shopping” phenomenon (Kasteler et al., 1976). However, physicians must accompany patients by explaining his/her clinical perspective and verifying that the choice is informed and legitimizing patients’ decisions, even if they differ from those that would guarantee clinical benefits.

Another ethical dilemma with a conflict between patient autonomy and beneficence is shown in Vignette 3. In this scenario, the physician exercises palliative paternalism with respect to the value of beneficence. Despite the patient’s contrariety stated in the documents, the physician performed the intervention, which was successful. The choice leads to the renunciation of the value of autonomy in the name of values of beneficence and non-maleficence. In such situations, it is important to consider whether the patient is able to choose, and appreciate the consequences of their choices, or whether the emotional suffering is excessive and significantly impedes the patient’s capacity to make particular medical decisions. The palliative paternalism led to a positive outcome in that it prevented the patient’s death.

**Vignette 3: Mr. LP**

A 44-year-old man suffering from chronic intestinal pseudo-obstruction (CIPO) for 13 years comes to the hospital in extreme prostration due to an acute episode requiring surgery. The patient is only partially able to interact due to severe distress and ongoing morphine treatment (chronically prescribed). The wife has always participated little in her husband’s albeit intense care due to her emotional fragility. She does not object to an emergency intervention that might resolve the situation. On the other hand, there is evidence in the patient’s documents of several conversations with the attending physician in which the patient claimed an unwillingness to further intensification of treatment, believing her life to be already very compromised. The physician explains to the patient that the intervention is very limited, emphasizing that there would be no substantial modification of the already extensive medical treatments thereafter (23 prescriptions per day, in addition to periodic bowel washings and intermittent fasting), and brings the patient into the room.

The surgery takes place successfully.

Under these circumstances, it is important to maintain a balanced approach that respects patient autonomy while allowing for professional guidance in complex end-of- treatment situations. The physician should guide the patient toward a decision that respects medical responsibilities and patient’s values. This balance can enhance patient care and ensure ethical integrity.

## 4 Discussions

Our aim was to discuss the main practical and theorical challenges that the physician-patient relationship undergoes in the context of end-of-treatment situations. We discussed the implications of end-of-treatment situations on clinical decision-making process and physicians’ role.

Patient-centered care advocates a personalization of the decision-making process, in which the physician’s approach varies according to the patient’s situation and individuality. However, some end-of-treatment situations, such as those presented, may lead to ethical conflicts, which become part of the decision-making process. In end-of-treatment situations in which the patient’s autonomy challenges proposed treatment, physicians should respect the patient’s autonomy and accept the patient’s choice to refuse treatment, even if this is deemed best for the patient’s health and in contrast with the value of beneficence. Conversely, in end-of-treatment situations where proposed (discontinuation of) treatment challenges the patient’s needs and preferences, a form of revised palliative paternalism may help to address the patient’s needs and support the decision as the treatment would cause more harm than benefits. The scenarios presented exemplify situations where ethical conflicts make it difficult to apply defined guidelines for best practice. Ethical conflicts must be managed with great attention to prevent them from influencing the decision-making process and leading to outcomes perceived as negative by healthcare professionals and/or patients. Jonsen et al. (2010) introduced the Four-Box Method to improve clinical ethics case analysis. The Four-Box Method is a structured framework for resolving ethical conflicts in end-of-treatment situations. It considers medical indications, patient preferences, quality of life, and contextual features. According to the authors, healthcare providers should systematically evaluate each of these aspects to better manage ethical dilemmas. Thus, physicians should identify the ethical issue. Then, they should gather all relevant information and discuss the benefit that the treatment/the intervention will provide toward the goal. They should identify the likelihood of success, the possible alternatives, and the potential harms of providing or not the treatment. This analysis should be done within the healthcare team and ethical committees so that there is a multidisciplinary and ethical perspective. Then, it is essential to discuss with patients and their family to consider how the clinical alternatives impact the patient’s daily life and think about the quality of life with and without treatment. The preferences of patients and their life context can be identified through the exploration of the patient’s agenda (Moja and Vegni, 2000). This exploration may also include the family system, especially in cases where there is direct involvement. Once the clinical situation is fully understood and the viable alternatives have been identified, it is helpful for healthcare professionals to discuss with the patient to ensure accountability and transparency. There are clinical situations in which the intervention of a third actor is needed; offering third-party insight can increase the objectivity of the assessment. At every stage, effective communication is essential, and conflict resolution strategies such as mediation and consensus-building techniques may facilitate the process.

By systematically evaluating each of these aspects, physicians can better manage ethical dilemmas.

The physician’s professional actions are always guided by ethical values; however, ethical values may change over time and with cultural progress. One of the most radical consequences of patient-centered care is the transformation of the idea of beneficence. In a disease-centered model, beneficence coincides with the care and treatment that best eradicate the disease and allow a prompt recovery. In contrast, in patient-centered care, beneficence is based on the bio-psycho-social concept of health, and considers medical and psychosocial circumstances, requiring a focus on the disease experience and the patient’s values. The principle of beneficence may therefore need to be revised in the patient care process and does not always coincide with biological health and treatment.

The decision-making process is often defined as a cognitive process, but several clinical circumstances make it clear that it also involves ethical and emotional dimensions. The decision-making process is influenced by values and emotions, especially those of patients (Leone et al., 2022). Often patients are not aware of their values and preferences until they enter a disease condition and are faced with difficult decisions. When preferences and principles are not specified in advance, patients may not be able to adopt a position of self-determination. In addition, emotional distress such as fear, angst, anger may significantly hinder patients’ ability to make informed decisions (Roeland et al., 2014). At the same time, the physicians’ psycho-emotional burden is noteworthy. Accepting a decision leading to the patient’s death is emotionally demanding and may lead to moral distress.

A transversal concept in clinical practice is the need for ongoing training for physicians. Adequate communication should characterize all stages of the relationship; however, it is crucial for ethical decision-making. It would therefore be appropriate to implement training courses, workshops, and seminars focused on the relational and ethical dimension of care to improve the healthcare professionals’ communication skills with the patient and within the team (Borghi et al., 2021).

Furthermore, due to the current paucity of end-of-treatment data, future studies should devote more attention to this complex phase and provide a better definition of end-of-treatment, especially in those contexts, such as medically assisted reproduction, where there is still a lack of agreement in this regard.

## Data Availability

The original contributions presented in the study are included in the article/supplementary material, further inquiries can be directed to the corresponding author.
